# The Reliability of the Resuscitation Assessment Tool (RAT) in Assessing Emergency Medicine Resident Competence in Pediatric Resuscitation Scenarios: A Prospective Observational Pilot Study

**DOI:** 10.7759/cureus.35869

**Published:** 2023-03-07

**Authors:** Meaghan J Mackenzie, Carly Hagel, Yiqun Lin, Andrew K Hall, Vincent J Grant, Shirmee Doshi

**Affiliations:** 1 Department of Emergency Medicine, University of Calgary, Calgary, CAN; 2 Department of Pediatrics, KidSIM Simulation Education and Research Program, Alberta Children's Hospital, Calgary, CAN; 3 Department of Community Health Sciences, University of Calgary, Calgary, CAN; 4 Department of Emergency Medicine, University of Ottawa, Ottawa, CAN; 5 Department of Emergency Medicine, Alberta Children's Hospital, Calgary, CAN

**Keywords:** resident doctor, emergency medicine resident, pediatric resuscitation, resuscitation assessment tool (rat), rat tool, medical education, competency by design, resuscitation, emergency medicine, simulation

## Abstract

Introduction

Emergency medicine (EM) postgraduate medical education in Canada has transitioned from traditional time-based training to competency-based medical education (CBME). In order to promote residents through stages of training, simulated assessments are needed to evaluate residents in high-stakes but low-frequency medical emergencies. There remains a gap in the literature pertaining to the use of evaluative tools in simulation, such as the Resuscitation Assessment Tool (RAT) in the new CBME curriculum design.

Methods

We completed a pilot study of resident physicians in one Canadian EM training program to evaluate the effectiveness and reliability of a simulation-based RAT for pediatric resuscitation. We recorded 10 EM trainees completing simulated scenarios and had nine EM physicians use the RAT tool to evaluate their performances. Generalizability theory was used to evaluate the reliability of the RAT tool.

Results

The mean RAT score for the management of pediatric myocarditis, cardiac arrest, and septic shock (appendicitis) across raters was 3.70, 3.73, and 4.50, respectively. The overall generalizability coefficient for testing simulated pediatric performance competency was 0.77 for internal consistency and 0.75 for absolute agreement. The performance of senior participants was superior to that of junior participants in the management of pediatric myocarditis (p = 0.01) but not statistically significant in the management of pediatric septic shock (p=0.77) or cardiac arrest (p =0.61).

Conclusion

Overall, our findings suggest that with an appropriately chosen simulated scenario, the RAT tool can be used effectively for the simulation of high-stakes and low-frequency scenarios for practice to enhance the new CBME curriculum in emergency medicine training programs.

## Introduction

Pediatric resuscitations are considered low-frequency, high-stakes events in emergency departments in North America [1,2]. One challenge is finding ways to provide emergency medicine (EM) residents with opportunities to demonstrate competence in this specific patient population without compromising safe patient care. The EM education community has recognized that simulation-based education can provide an alternative way to expose residents to rare presentations in a controlled setting [3,4] as well as allow for assessment of competence.

Post-graduate medical education in Canada is in a state of transition from traditional time-based training to competency-based medical education (CBME). As opposed to determining competency based on the number of years of training, CBME aims to do so by having residents prove proficiency in a set number of clinical skills or tasks. The Royal College of Physicians and Surgeons of Canada has implemented Competence by Design (CBD), its own unique model of CBME [5]. The foundation of the program is the assessment of entrustable professional activities (EPAs) [6]. EPAs are defined as "units of professional practice that can be fully entrusted to a trainee once he or she has demonstrated the necessary competence to execute this activity unsupervised" [7]. Assessments of performance on EPAs are designed to be low-stakes but high-frequency assessments to track the achievement of competencies over time. Trainees are promoted to advanced stages of training by demonstrating competence in stage-specific EPAs.

The transition to CBME in emergency medicine in Canada was in July 2018 [8]. This transition requires EM residents to achieve competence in a number of EPAs during each stage of training. Competence in managing pediatric resuscitation is an expectation at all levels of training, with multiple assessments required.

While many assessment tools for EM simulation scenarios have been created and arguments for their validity presented, a limited number have been integrated into the EM residency curriculum as a form of assessment of competence [9]. With the rollout of CBME in EM, programs are facing an increased need to use simulation to provide residents with exposure to uncommon clinical scenarios. EPAs have primarily been designed for the clinical setting, and the assessment guidelines for each EPA are not specific to simulated practice scenarios [5]. Currently, programs are using assessment tools that have not yet been validated for use in simulation to provide assessment data that can influence the progress of trainees.

Queens University (Kingston, Ontario, Canada) created a resuscitation assessment tool (RAT) with the goal of utilizing it within the new CBD curriculum. The RAT generates entrustment scores through an anchored global assessment scale. Recently, the research team from Queens University that created the RAT used it to compare resident performance in the clinical environment relative to the simulated (SIM) environment [10]. Using the RAT, they were able to identify residents who needed support in specific competencies or areas.

There remains a gap in the literature on the use of tools such as the RAT in the new CBME curriculum design, specifically pertaining to the assessment of competence in pediatric resuscitation and pediatric EPAs. There are specified performance expectations for a required number of exposures to pediatric acute care cases, with accompanying assessments of competency in these settings. Additionally, while there is a clearly established transferability from simulated practice to clinical competence for procedural skills [11], that same link has not yet been well established for resuscitation competencies [12].

Our study aims to evaluate the reliability of the RAT as a tool for assessing emergency medicine trainee competence, specifically in pediatric resuscitation scenarios. We aim to achieve this by determining whether the RAT can communicate consistent results independent of the assessor (rater) and if it can correctly identify residents who may need more support in a specific area of competence. The overarching goal of this study is to understand how to strengthen pediatric simulated curricula for EM residents participating in the CBME framework.

## Materials and methods

A prospective observational cohort study of resident physicians in one Canadian EM training program was used to evaluate the effectiveness and reliability of a simulation-based RAT for pediatric resuscitation. The study took place between January 2019 and July 2021 and was carried out in the KidSIM Lab at the Alberta Children’s Hospital in Calgary, Alberta, Canada. The study was approved by the Conjoint Health Research Ethics Board at the University of Calgary.

Study setting

The study took place at the University of Calgary’s Cumming School of Medicine Department of Emergency Medicine over an 18-month period of time. High-fidelity and age-appropriate mannequins were utilized in the KidSIM Center at the Alberta Children’s Hospital. Performance was video recorded from three fixed camera angles to allow adequate views of the trainee, the mannequin, and the monitors.

Study participants

Emergency medicine residents (FRCPC-EM), pediatric emergency medicine fellows, and family medicine residents completing a special competence year in emergency medicine (CCFP-EM) were eligible to participate. We recruited 10 residents, as determined by local resident volume and availability. Participation was voluntary, and residents were assured that their performance would not be shared with anyone outside of the study (program directors or otherwise). They were informed, prior to consenting to participate, that there would be raters from within Calgary as well as across the country. Written consent was obtained from all participants, including video recordings of their performances in the simulation lab.

Assessor training

A purposeful sample of practicing EM physicians involved in CBME simulation training for residents was chosen as raters. The authors utilized frame-of-reference training to prepare raters and provided an orientation to the RAT tool prior to reviewing each resident’s performance. Each rater received a practice video of a resident physician not participating in the study completing a simulated scenario. The rater was required to watch the video and use the RAT tool to evaluate the performance. After the practice video, a member of the research team (MM) met with each participant over a 30-minute period to go over their evaluation, compare RAT evaluations, and discuss any questions that came up about the tool. Raters were then asked to rate the performance of the study participants in all three scenarios, as detailed below.

A total of nine EM physicians were trained as assessors: three of those physicians were local and unblinded, and six were external EM physicians from across Canada who were blinded to each resident’s level of training. Apart from EM training, all raters had specific interests and training in simulated practice, curriculum design, and residency education.

Each EM physician rated four residents for a total of twelve total evaluations. Each resident was reviewed by one unblinded rater and two blinded raters. Raters were blinded to each resident's level of training. Blinded as well as unblinded raters were recruited to ensure that scores were not biased by the rater's awareness of the resident’s year of training. To further assess any inconsistencies in rating, all nine EM assessors rated one of the residents.

Measures (RAT)

**Figure 1:**
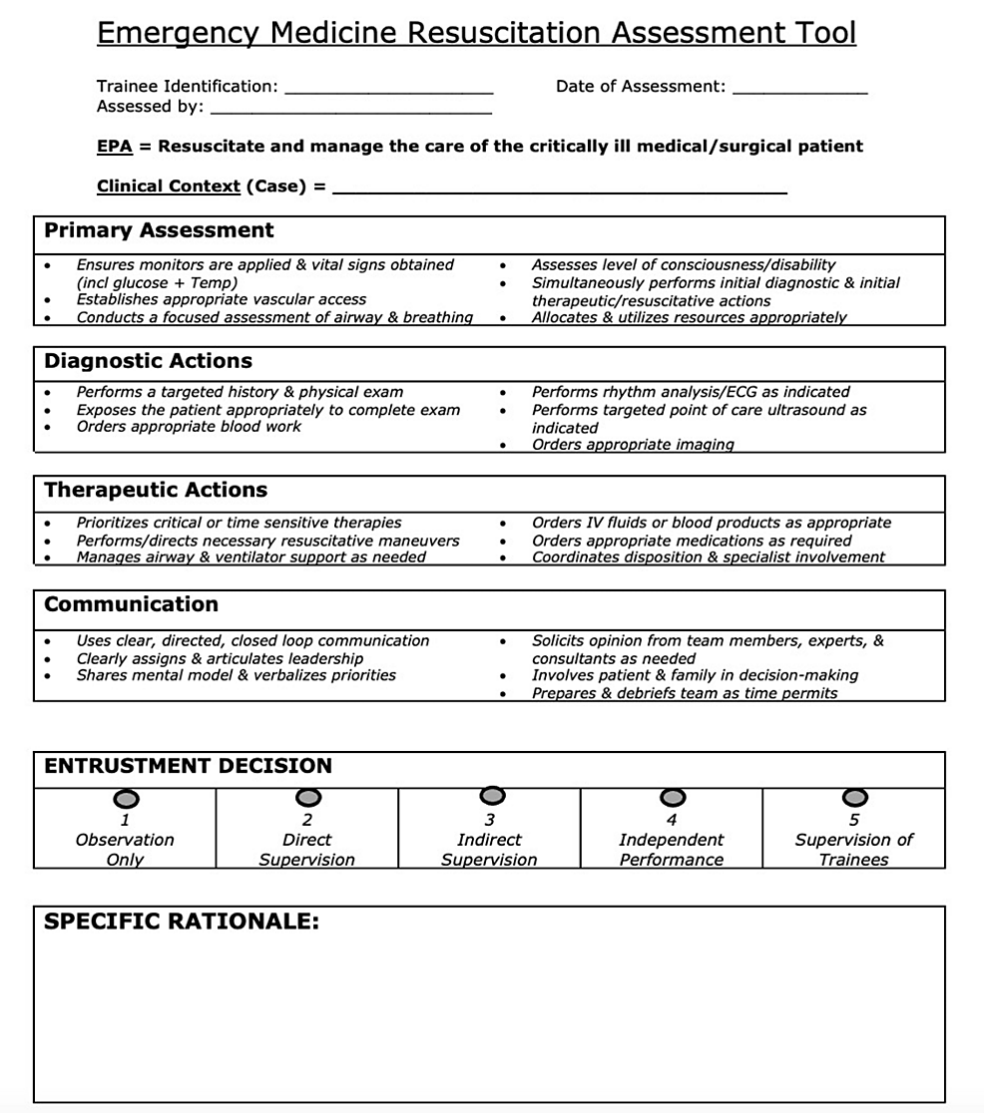
Emergency medicine resuscitation assessment tool

The RAT was derived from a prior assessment tool, the Queen’s simulation assessment tool (QSAT). The QSAT was recently modified in the context of the implementation of CBME to create the resuscitation assessment tool (RAT), with the goal of utilizing it within the new CBD curriculum. The RAT generates entrustment scores through an anchored global assessment scale. The scoring system was derived from the QSAT by developing generic behavioral anchors for resuscitation performance using a modified Delphi process for each domain and replacing the global assessment scale with an entrustment scale. A score of one on the scale translates to "resident only observed the skill," whereas a score of five communicates that the resident has mastered the skill and can supervise trainees (Figure 1).

Simulated scenario administration and evaluation

Each resident completed three standardized simulated cases in the simulation labs. They were each assigned the role of team leader. As described on the Royal College website, the goal of this EPA is to lead a team of healthcare professionals in the emergency department to care for a patient with a medical or surgical life-threatening condition. Their performances were video recorded for later review. KidSIM nurses supported and facilitated the scenarios, keeping each scenario consistent for each resident. Though all the staff was experienced with the mannequins and the scenarios, we reviewed each scenario 30 minutes prior to each simulation session. There were two simulation days, and each day the same KidSIM staff served as facilitators. The scenarios were based on EPA 3.1: resuscitating and coordinating care for critically ill patients (focused on cardiorespiratory arrest, dysrhythmias, shock, respiratory distress, or altered mental status). Our cases included pediatric myocarditis, septic shock (appendicitis), and cardiac arrest. Details on these case scenarios can be found in the appendices.

Statistical analysis

Demographic characteristics of the participants, such as gender and level of training, were summarized with descriptive statistics (count and percentage). The RAT scores of the three scenarios were summarized with a mean and standard deviation.

**Figure 2:**
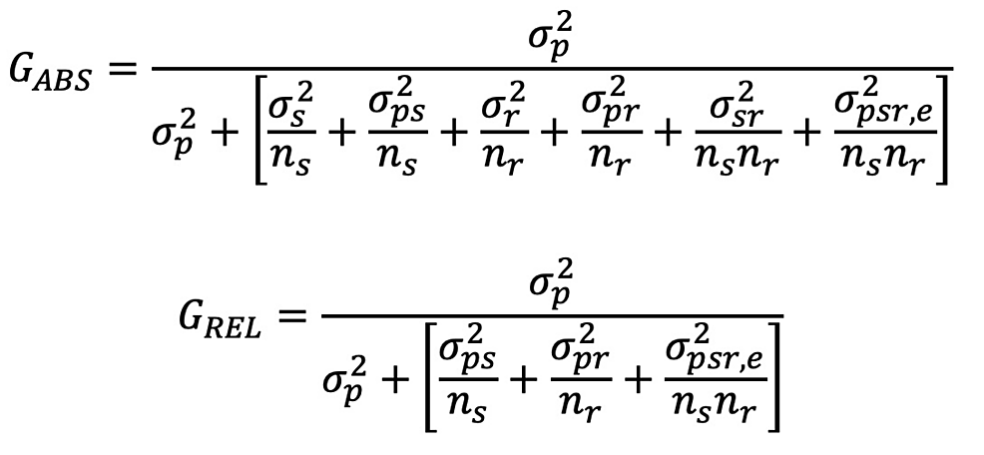
A two-random facet design where both the effects of the rater and scenario are considered random

**Figure 3:**
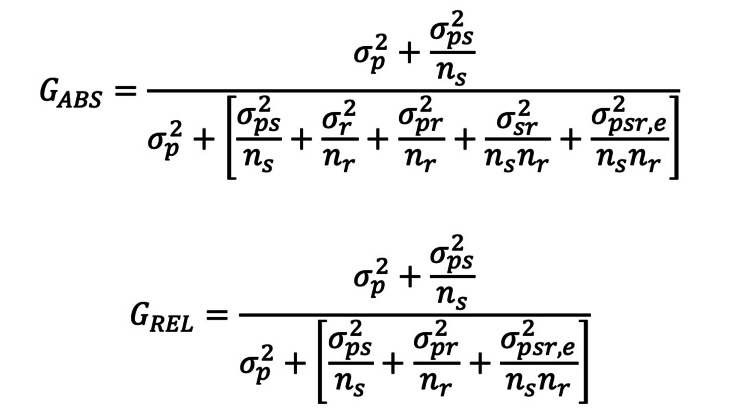
A two-facet design with one facet fixed where the facet of the rater was considered random and the facet of the scenario was considered fixed

Generalizability theory was used to evaluate the reliability of the RAT tool. We used R statistical software (www.r-project.org) and "gtheory" packages to estimate the variance components of participants, scenarios (i.e., pediatric cardiac arrest, myocarditis, septic shock (appendicitis), and raters. The three two-way interactions between all three of these variables (raters x scenarios, raters x participants, scenario x participants) and the three-way interaction effects (raters x participants x scenarios) were confounded with random error as a function of the fully crossed design. The generalizability coefficients for internal consistency and absolute agreement of the following two analyses were calculated manually:

(1) A two-random-facet design where both the effects of the rater and scenario are considered random (Figure 2).

(2) A two-facet design with one facet fixed. The facet of the rater was considered random, and the facet of the scenario was considered fixed since the same scenario could be used to evaluate the resident’s performance (Figure 3).

The variance coefficients were then used to inform a decision study (D-study) to determine the optimal combination of raters and scenarios to maximize the reliability of the assessments.

Due to the small sample size and skewed distribution of the data, non-parametric tests were used for validity tests. The Wilcoxon rank sum test was used to compare the performance of junior (post-graduate years one and two) and senior participants (post-graduate years three, four, and five) in the three scenarios. The Wilcoxon signed-rank tests were used to compare the performances within three scenarios. The RAT score was the only primary outcome we examined. The structure of analysis plan was organized as (1) demographic information, (2) generalizability theory (reliability), and (3) validity.

## Results

Participant demographics

A total of 10 emergency medicine residents participated in the study, with four women (4/10, 40%) and six men (6/10, 60%). Eight participants were emergency medicine residents (FRCPC-EM) from post-graduate year one to five (PGY-1: 1/10, PGY-2: 3/10, PGY-3: 3/10, PGY-5: 3/10), one was a pediatric emergency medicine fellow (PGY-4), and one was a former family medicine resident completing a year of emergency medicine training (CCFP-EM) (PGY-3).

Data summary

The mean RAT score for the three pediatric simulated resuscitation performances across participants and raters was 3.97. The mean score for the management of pediatric myocarditis, cardiac arrest, and septic shock (appendicitis) across raters was 3.70, 3.73, and 4.50, respectively. Table 1 provides a summary of mean RAT scores for juniors (PGY-1 or 2), seniors (PGY-3 or above), and total participants in three scenarios.

**Table 1:** Summary of resuscitation assessment scores in three scenarios Data presented as mean ± standard deviation Junior: post-graduate years one and two emergency medicine resident; Senior: post-graduate years three to five emergency medicine resident

Scenario	Total	Junior	Senior
Myocarditis	3.70 ± 0.84	3.20 ± 0.68	4.20 ± 0.68
Cardiac arrest	3.73 ± 0.78	3.67 ± 0.82	3.80 ± 0.77
Septic shock (appendicitis)	4.50 ± 0.63	4.47 ± 0.64	4.53 ± 0.64

Generalizability study

The variance components for testing the participants across three pediatric resuscitation scenarios and 10 raters are presented in Table 2. The overall generalizability coefficient for testing simulated pediatric performance competency was 0.77 for internal consistency and 0.75 for absolute agreement for the 10-rater, three-fixed scenario design. If ignoring the variance components of scenarios, the generalizability coefficient was 0.00, as shown in Table 3.

**Table 2:** Variance by source Df: degrees of freedom; Sum Sq: sum of squares; Mean Sq: mean square *The estimated variances were shown at three decimal places, not exactly 0 The estimated variance for a resident was 1.1 x 10-9. The estimated variance for the rater was 3.9 x 10 -11

	Df	Sum Sq	Mean Sq	Estimated variance	Variance percent (%)
Resident	9	8.926	0.992	0.000*	0.0
Rater	8	2.963	0.370	0.000*	0.0
Scenario	2	15.167	7.584	0.163	21.4
Resident × Rater	18	10.444	0.580	0.076	10.0
Resident × Scenario	18	17.574	0.976	0.186	24.5
Rater × Scenario	16	8.537	0.534	0.053	7.0
Resident × Rater × Scenario, error	36	10.056	0.279	0.282	37.1

**Table 3:** Reliability coefficients σ^2^: relevant variance; σ^2^: absolute variance; G-Rel: relevant G coefficient; G-Abs: absolute G coefficient

Model	σ^2^-Rel	σ^2^-Abs	G-Rel	G-Abs
2 random facet	0.081	0.137	0.000	0.000
1 random (rater), 1 fixed (scenario) facet	0.081	0.083	0.766	0.748

Decision study

A decision study (D-study) was conducted as a hypothetical calculation whereby the number of raters and scenarios were manipulated to achieve a reliability coefficient of 0.70. We found that increasing the number of scenarios did not seem to improve the reliability coefficient. Based on the hypothetical projection of the D-study, two scenarios and six raters would be sufficient to achieve a reliability coefficient of 0.70 (Table 4).

**Table 4:** Generalizability and decision study results for various combinations of scenarios and raters G-Rel: relevant G coefficient; G-Abs: absolute G coefficient

Number of scenarios	Number of raters	G-Rel	G-Abs
2	8	0.78	0.75
2	7	0.76	0.73
2	6	0.73	0.70
2	5	0.69	0.66
4	8	0.73	0.71
4	7	0.70	0.68
4	6	0.67	0.65

There was an agreement between blinded and unblinded evaluators throughout each scenario.

Validity evidence

**Figure 4:**
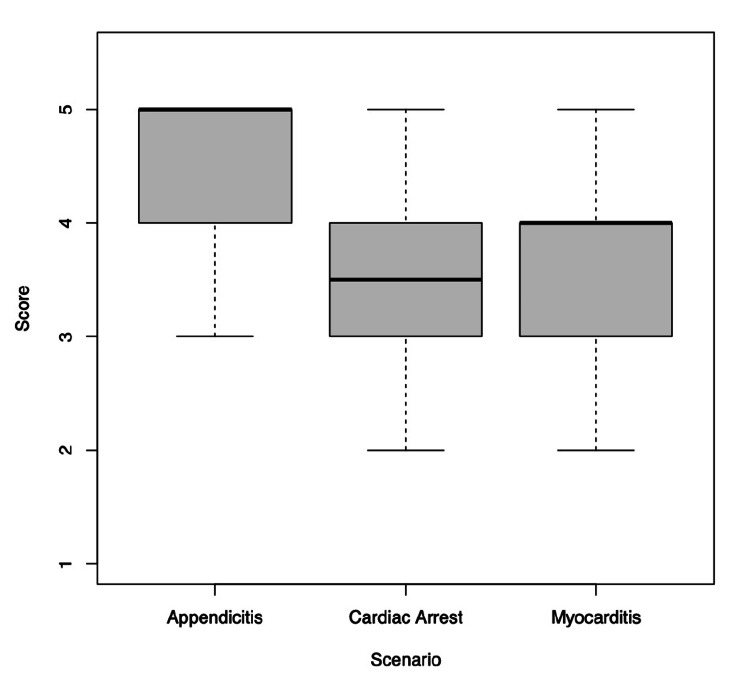
The rating scores for three simulated scenarios

The performance of senior participants was superior to that of junior participants in the management of pediatric myocarditis (junior vs. senior median score: 3 vs. 4, p = 0.01). The performance difference in the management of cardiac arrest and septic shock (appendicitis) didn’t yield statistical significance (septic shock: p = 0.77; cardiac arrest: p = 0.61). We found that across all levels of training, residents performed better on the septic shock (appendicitis) case and consistently scored lower on the myocarditis and cardiac arrest cases (septic shock vs. cardiac arrest/myocarditis median score: 5 vs. 4, p = 0.01) (Figure 4).

## Discussion

Our findings suggest that the RAT tool can be used reliably for the simulation of pediatric resuscitation scenarios as a practice to enhance the CBME curriculum. The tool was found to be reliable in this context, where raters are trained and calibrated appropriately to properly use the tool using a frame of reference approach. It was able to show good agreement between multiple raters and therefore can be trusted to give residents consistent results. The variance between raters was 0.0% (Table 2). An agreement amongst raters is necessary when using a tool to evaluate residents and promote them to new stages of training based on simulated performance.

Simulation-based education provides many advantages, including exposure to infrequent but critical clinical scenarios [13,14], the opportunity to record and review sessions to provide more robust feedback, and the ability to assess teamwork, communication, and crisis resource management skills. This type of education is particularly useful in pediatric resuscitations, which are low-frequency, high-stakes events. For simulation to be utilized effectively for assessment, there are many considerations, including the reliability and validity of the assessment tools and processes, the relevance of outcome measures, feasibility, and rater training [15].

The RAT was also able to reliably identify pediatric resuscitation topics and themes that residents struggled with and could therefore be used to determine the content for future simulations. Residents consistently scored higher on the septic shock (appendicitis) case and lower on the myocarditis and cardiac arrest cases (Figure 4). This was independent of training level. There was a great degree of variance among scenarios (21.4%), which reflects performance differences based on the scenario itself. Senior residents consistently scored higher than junior residents across all scenarios, with the largest gap in the myocarditis case (Table 1). Of note, in the myocarditis case, the performance of the senior residents was superior with statistical significance (junior vs. senior median score: 3 vs. 4, p=0.01). However, in the cardiac arrest and septic shock cases, the difference was not statistically significant. This was likely the case for a few reasons, the first of which was the small sample size. This study was designed for generalizability theory (for reliability), not for establishing discriminant validity evidence. The other contributor was likely the difficulty and complexity of each scenario. If cases are too difficult or too easy for participants, it makes it more difficult to differentiate participants. It is postulated by the authors that the decrease in performance related to the management of pediatric myocarditis, as compared to pediatric septic shock, is likely because residents are less familiar with more severe and uncommon pediatric presentations. This simulated scenario has been used for resident training in the KidSIM lab previously without prior identification of an issue with the scenario itself. However, another potential confounder that could have influenced the difference in score could have been the specific delivery of the case, although we followed the scenario card in a precise fashion. Altogether, this highlights that in order to keep simulated practice high yield, case selection must be done carefully to highlight cases that learners encounter less frequently. Competency in these cases should be achieved prior to being confidently promoted to the next stage in training.

Limitations

Despite providing support for the use of the RAT tool in the emergency medicine CBD curriculum, there are a few noteworthy limitations that deserve mention in our study. Given the small sample size, the variability in resident scores may be overstated, and the generalizability of our results should be taken in context. While the low number of participants is a limitation, the lack of variance among our residents and raters gives important information on the structure of simulated scenarios moving forward. Future work with a variety of challenging cases would be important to assess the variance between learners based on experience and skill level.

The authors were initially concerned that the data collection could also carry inherent bias. We recruited three local raters, all of whom knew the residents they were rating. This could have created a bias in the scores given. In contrast, blinded external raters were also used, and they were found to have an agreement with the local raters (Table 2). Unfortunately, a blinded rating is not possible in the clinical environment, even in simulated practice, due to logistical constraints.

## Conclusions

The RAT was found to be reliable when raters are appropriately trained and there is good agreement with zero variance between multiple raters. Therefore, the RAT can be trusted to give residents consistent evaluations. Additionally, the tool was able to reliably identify resuscitation topics that residents struggled with, which will help educators choose content for future simulated scenarios.

Finally, we also found that the resident level of training and rater blinding did not impact the reliability of the tool. Overall, this study demonstrates that the RAT is a reliable tool to implement in the evaluation of emergency medicine residents for pediatric resuscitation scenarios in a simulation curriculum within CBD training.

## Figures and Tables

**Figure 5:**
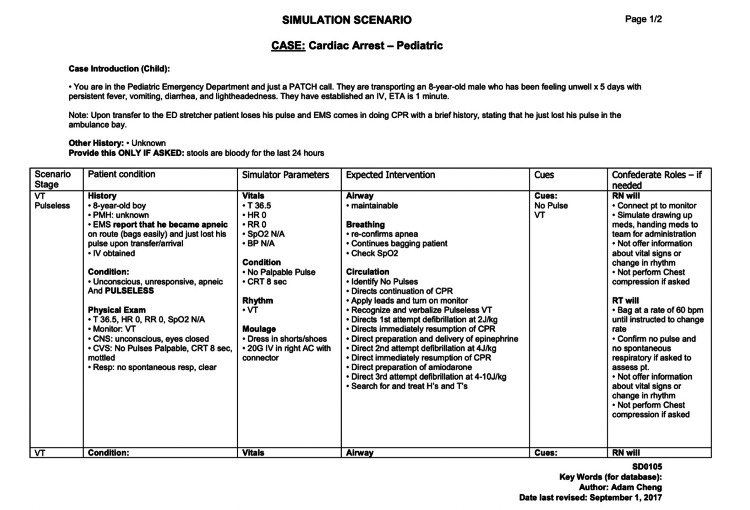
Cardiac arrest simulation scenario (part 1 of 2)

**Figure 6:**
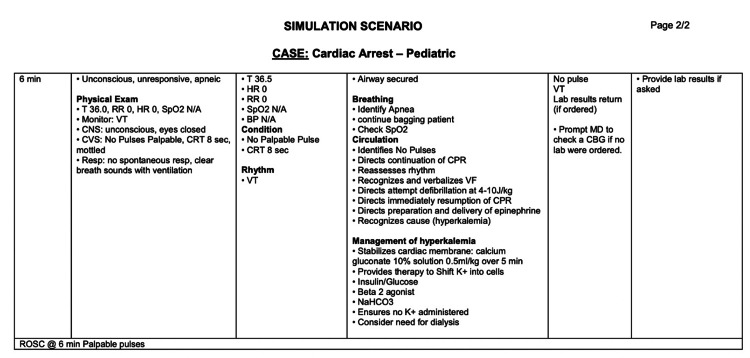
Cardiac arrest simulation scenario (part 2 of 2)

**Figure 7:**
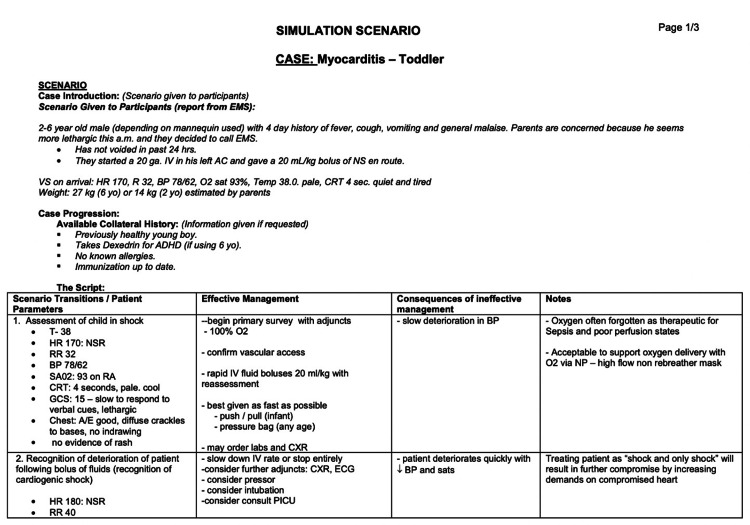
Myocarditis simulation scenario (part 1 of 3)

**Figure 8:**
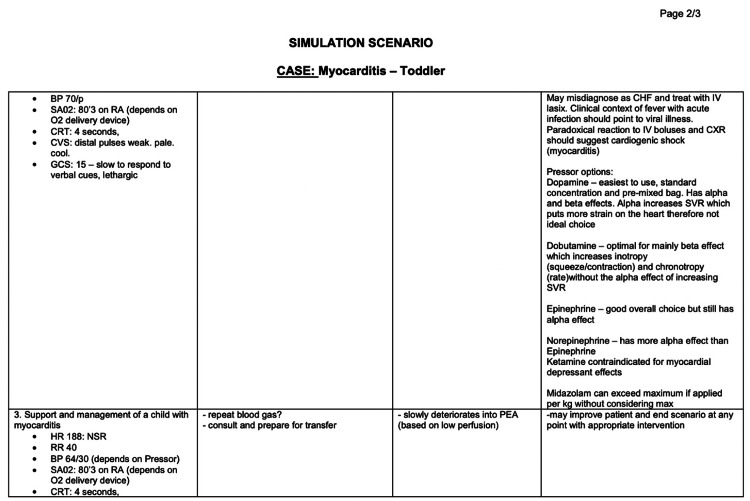
Myocarditis simulation scenario (part 2 of 3)

**Figure 9:**
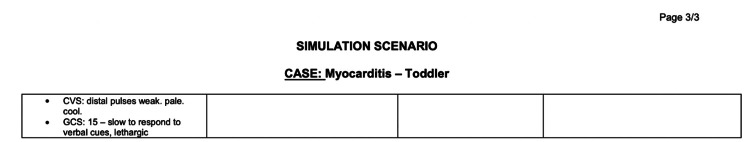
Myocarditis simulation scenario (part 3 of 3)

**Figure 10:**
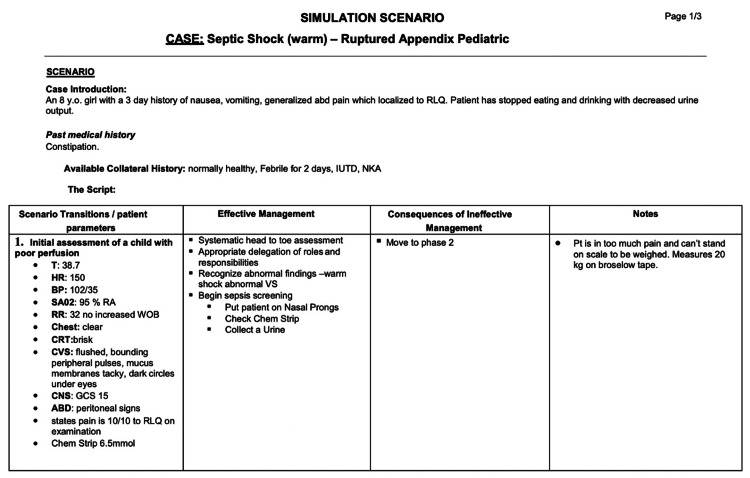
Septic shock due to appendicitis simulation scenario (part 1 of 3)

**Figure 11:**
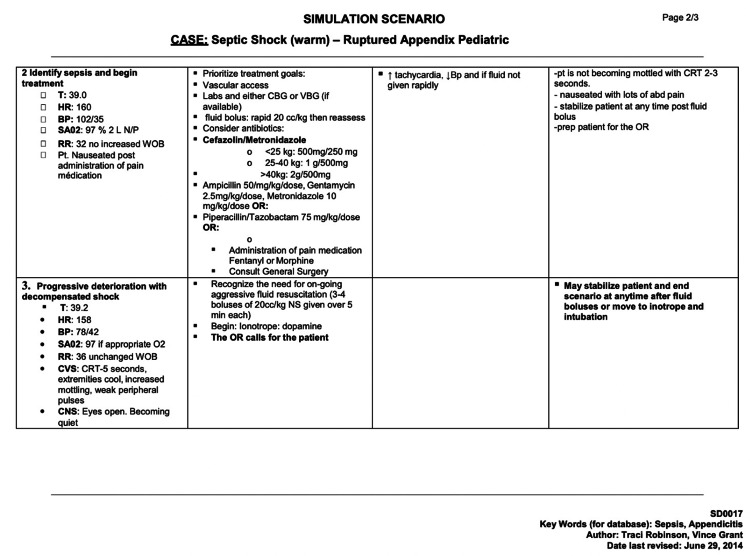
Septic shock due to appendicitis simulation scenario (part 2 of 3)

**Figure 12:**
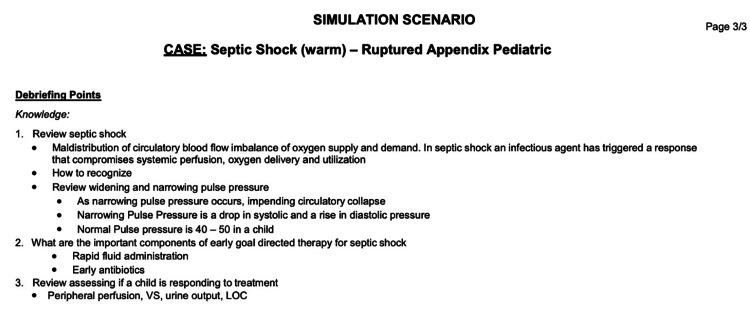
Septic shock due to appendicitis simulation scenario (part 3 of 3)

## Appendices

The three simulation scenarios used to assess the reliability of the RAT tool were cardiac arrest (Figures 5, 6), myocarditis (Figures 7-9), and septic shock due to appendicitis (Figures 10-12).
